# Molecular Responses of Human Retinal Cells to Infection with Dengue Virus

**DOI:** 10.1155/2017/3164375

**Published:** 2017-11-12

**Authors:** Jillian M. Carr, Liam M. Ashander, Julie K. Calvert, Yuefang Ma, Amanda Aloia, Gustavo G. Bracho, Soon-Phaik Chee, Binoy Appukuttan, Justine R. Smith

**Affiliations:** ^1^Microbiology & Infectious Diseases, Flinders University School of Medicine, Rm 5D-316, 1 Flinders Drive, Bedford Park, Adelaide, SA 5042, Australia; ^2^Eye & Vision Health, Flinders University School of Medicine, Rm 4E-431, 1 Flinders Drive, Bedford Park, Adelaide, SA 5042, Australia; ^3^Flinders Centre for Innovation in Cancer, Flinders University School of Medicine, 1 Flinders Drive, Bedford Park, Adelaide, SA 5042, Australia; ^4^Ocular Inflammation and Immunology Service, Singapore National Eye Centre, 11 Third Hospital Avenue, Singapore 168751

## Abstract

Recent clinical reports indicate that infection with dengue virus (DENV) commonly has ocular manifestations. The most serious threat to vision is dengue retinopathy, including retinal vasculopathy and macular edema. Mechanisms of retinopathy are unstudied, but observations in patients implicate retinal pigment epithelial cells and retinal endothelial cells. Human retinal cells were inoculated with DENV-2 and monitored for up to 72 hours. Epithelial and endothelial cells supported DENV replication and release, but epithelial cells alone demonstrated clear cytopathic effect, and infection was more productive in those cells. Infection induced type I interferon responses from both cells, but this was stronger in epithelial cells. Endothelial cells increased expression of adhesion molecules, with sustained overexpression of vascular adhesion molecule-1. Transcellular impedance decreased for epithelial monolayers, but not endothelial monolayers, coinciding with cytopathic effect. This reduction was accompanied by disorganization of intracellular filamentous-actin and decreased expression of junctional molecules, zonula occludens 1, and catenin-*β*1. Changes in endothelial expression of adhesion molecules are consistent with the retinal vasculopathy seen in patients infected with DENV; decreases in epithelial junctional protein expression, paralleling loss of integrity of the epithelium, provide a molecular basis for DENV-associated macular edema. These molecular processes present potential therapeutic targets for vision-threatening dengue retinopathy.

## 1. Introduction

Human infection with dengue virus (DENV) is transmitted by *Aedes* species mosquitos and presents a broad spectrum of disease, ranging from subclinical to “severe” dengue, which is characterized by life-threatening plasma leakage, hemorrhage, and/or organ failure [1, 2]. Recent research, involving sophisticated statistical modeling of data derived from over 8000 occurrence records, predicts that there are approximately 390 million DENV infections worldwide each year [3]. The Global Burden of Disease Study 2015 [4] has highlighted DENV infection as an exception to the general trend for falling mortality rates related to neglected tropical diseases: between 2005 and 2015, the number of deaths from DENV infection worldwide rose by almost 50% from 12,300 to 18,400.

Epidemics of virologically confirmed DENV infection are on record from the 1940s [5], but there was little recognition of dengue eye disease until the 2000s. Multiple forms of dengue eye disease have been reported recently, affecting the orbit, ocular surface, and/or intraocular tissues [6]. Intraocular manifestations, particularly those that involve the retina, are well described and are most likely to adversely impact the vision. Dengue retinopathy may take the form of a retinal vasculopathy, with clinically apparent or presumed subclinical retinal vasculitis, retinal hemorrhage, and/or vascular occlusion [7–9]. This vasculopathy preferentially affects the central macular region of the retina, but other macular involvements are also observed. Macular edema is the most prevalent form of maculopathy; another maculopathy, which is termed “foveolitis,” is less common, but characteristic of dengue retinopathy, and diagnosed on the basis of a yellow-orange dot in the macula that has been localised to the border of the neuroretina and retinal pigment epithelium by ophthalmic imaging [10–13]. Choroidal neovascularization at the macula is also possible [14]. The prognosis of dengue retinopathy is highly variable, ranging from full resolution to permanent vision loss, irrespective of medical interventions to reduce inflammation [6].

While cellular and molecular mechanisms of systemic dengue have been extensively investigated, the basic processes that contribute to dengue retinopathy remain unstudied. We have initiated this investigation by studying interactions between DENV and human retinal endothelial cells and retinal pigment epithelial cells, using established cells lines and primary cells, and laboratory and patient DENV isolates. Our rationale for focusing on these cell subpopulations was twofold. Firstly, retinal endothelial cells and retinal pigment epithelial cells constitute the blood-retinal barrier [15], and therefore they are the first cells DENV encounters when entering the retina. Secondly, clinical manifestations in patients [8–14]—retinal vasculopathy and maculopathy—implicate these cell subtypes in the ocular pathology. We present observations relating to the susceptibility of the cells to infection with DENV, the type I interferon (IFN) antiviral and inflammatory responses of DENV-infected cells, and the impact of DENV infection on barrier function of the cells.

## 2. Materials and Methods

### 2.1. Human Ocular Cell Lines

Primary human retinal cells were isolated from cadaver donors obtained from the Eye Bank of South Australia (Adelaide, Australia) within 24 hours of death with the approval of the Southern Adelaide Clinical Human Research Ethics Committee.

To isolate primary human retinal pigment epithelial cells, the method published by Blenkinsop et al. [16] was followed, with some modifications. In brief, choroid with adherent retinal pigment epithelium was dissected from posterior eyecups and digested with 0.5 mg/mL collagenase IA and 0.5 mg/mL collagenase IV solution (Sigma-Aldrich, St. Louis, MO). Retinal pigment epithelial cells were separated from choroid as sheets in phosphate buffered saline (PBS) with 2% fetal bovine serum (FBS, Bovogen Biologicals, Keilor East, Australia, or GE Healthcare-HyClone, Logan, UT) and layered over 20% sucrose in medium. Cells were cultured in Dulbecco's modified Eagle's medium : nutrient mixture F12 (DMEM : F12, Thermo Fisher Scientific-Gibco, Grand Island, NY) and minimum essential medium Eagle (MEM, Sigma-Aldrich), in a ratio of 1 : 1, supplemented with FBS (initially at 10%, reduced to 2% after 2 days), 1x N1 Medium Supplement, 0.25 mg/mL taurine, 0.02 mg/mL hydrocortisone, and 0.013 ng/mL triiodothyronine (all from Sigma-Aldrich), and 1x MEM Non-Essential Amino Acids Solution, 1x GlutaMAX Supplement, and 100 U/mL penicillin-100 *μ*g/mL streptomycin (all from Thermo Fisher Scientific-Gibco) at 37°C and 5% CO_2_ in air.

To isolate primary human retinal endothelial cells, we followed the method we have previously published [17]. Retina was dissected from posterior eyecups, and digested with 0.25 to 1 mg/mL type II collagenase (Thermo Fisher Scientific-Gibco). After 7 days of culture in MCDB-131 medium (Sigma-Aldrich) with 2% FBS and endothelial growth factors (EGM-2 SingleQuots supplement, omitting FBS, hydrocortisone and gentamicin; Clonetics-Lonza, Walkersville, MD), endothelial cells were purified using Dynal magnetic beads (Thermo Fisher Scientific-Invitrogen, Oslo, Norway) coated with mouse monoclonal anti-human CD31 antibody (BD Biosciences-Pharmingen, San Diego, CA), and grown in modified MCDB-131 medium with 10% FBS at 37°C and 5% CO_2_ in air.

The ARPE-19 human retinal pigment epithelial cell line (American Type Culture Collection (ATCC), Manassas, VA) [18] was cultured in DMEM : F12 supplemented with 5% FBS at 37°C and 5% CO_2_ in air. The human retinal endothelial cell line was generated and characterized in-house, as we have reported in detail [17]. In brief, endothelial cells, isolated as described above, were expanded by transduction with the mouse recombinant amphotropic retrovirus, LXSN16E6E7 (gifted by Denise A. Galloway, PhD, Fred Hutchinson Cancer Institute Seattle, WA) [19]. This cell line retains an endothelial phenotype, including expression of endothelial markers and formation of capillary-like tubes on basement membrane substitute [17]. Retinal endothelial cells were cultured in MCDB-131 medium supplemented with 5% FBS and endothelial growth factors at 37°C and 5% CO_2_ in air.

### 2.2. Dengue Virus

The DENV strains included Mon601 (full-length, infectious recombinant clone of DENV serotype 2 (DENV-2) New Guinea C strain, originally cloned from a mouse brain-adapted isolate) [20] and PUO-312 (DENV-2 strain isolated in the field during the 1980 Thai dengue epidemic, and provided by Peter J. Wright, PhD (Monash University, Melbourne, Australia) [21, 22]. Both Mon601 and PUO-312 strains have tropism for human cell lines and primary cells [23–25]. The Mon601 strain virus was generated by transfection of *in vitro* transcribed DENV RNA into baby hamster kidney BKH-21 fibroblasts and amplified in C6/36 mosquito cells. The PUO-312 strain virus was continuously propagated in C6/36 mosquito cells. Virus stocks were titrated by plaque assay on Vero cells (ATCC), with plaques detected by neutral red overlay, and expressed as plaque-forming units (pfu)/mL.

### 2.3. Viral Infection of Human Ocular Cells

Unless otherwise stated, retinal cells were plated for confluence on surfaces appropriate to the assay in modified DMEM : F12 or modified MCDB-131 medium, respectively, and incubated overnight at 37°C and 5% CO_2_ in air. Cell monolayers were inoculated with DENV at multiplicity of infection of 1, or mock-infected, in FBS-free medium for 90 minutes with intermittent rocking, washed 3 times with fresh FBS-free medium, and returned to modified DMEM : F12 or modified MCDB-131 medium for incubations of up to 72 hours postinoculation. Viral titer in culture supernatant was determined by plaque assay, as described above.

### 2.4. Immunocytochemistry

Confluent monolayers of ARPE-19 retinal pigment epithelial cells or retinal endothelial cells on gelatin-coated glass coverslips, infected with DENV-2 or mock-infected, were fixed in 4% paraformaldehyde, rinsed in 70% ethanol, and stored in sterile PBS. Monolayers were permeabilized with 0.05% IGEPAL CA-630 (Sigma-Aldrich) and blocked in 4% goat serum (Thermo Fisher Scientific-Gibco), 5% FBS, and 0.4% bovine serum albumin (Sigma-Aldrich) in PBS. Cells were labeled with mouse anti-double-stranded RNA (dsRNA) monoclonal antibody (SCICSONS, Budapest, Hungary: described, J2) diluted to 2.5 *μ*g/mL, or serum from a DENV-infected human (gift of Dr. Wright) diluted 1 in 3000, both in PBS with 2% goat serum, for 60 minutes at room temperature, and subsequently washed in PBS. Cells were incubated with Alexa Fluor 488-tagged goat anti-mouse IgG or Alexa Fluor 555-goat anti-human IgG (Thermo Fisher Scientific-Molecular Probes, Eugene, OR) at 2 *μ*g/mL in PBS with 2% goat serum for 30 minutes at room temperature. After washing with PBS, cells were counterstained with Hoechst 33342 nucleic acid stain (Thermo Fisher Scientific-Molecular Probes) for 10 minutes. Monolayers were mounted in ProLong Gold Antifade Mountant (Thermo Fisher Scientific-Molecular Probes) and imaged by confocal microscopy (Leica TCS SP5 Confocal Microscope: Leica Microsystems, Mannheim, Germany).

### 2.5. RNA Isolation, Reverse Transcription, and Polymerase Chain Reaction

At the conclusion of infections, total RNA was extracted from TRIzol Reagent (Gibco)-lysed cells, according to the manufacturer's instructions, and stored at −80°C ahead of the reverse transcription (RT). RNA concentration was determined by spectrophotometry on the NanoDrop 2000 instrument (Thermo Fisher Scientific, Wilmington, DE). For examination of the host cell response to infection, reverse transcription was performed using the iScript Reverse Transcription Supermix for RT-qPCR (Bio-Rad, Hercules, CA), with 500 ng of RNA template yielding 20 *μ*L cDNA. Quantitative real-time PCR was performed on the CFX Connect Real-Time PCR Detection System (Bio-Rad) using the following: 2 *μ*L of cDNA, diluted 1 in 10; 4 *μ*L of iQ SYBR Green Supermix; 1.5 *μ*L each of 20 *μ*M forward and reverse primers; and 11 *μ*L of nuclease-free water for each reaction. Amplification consisted of the following: a precycling hold at 95°C for 5 minutes; 40 cycles of denaturation for 30 seconds at 95°C; annealing for 30 seconds at 60°C; extension for 30 seconds at 72°C; and a postextension hold at 75°C for 1 second. A melting curve, representing a 1-second hold at every 0.5°C between 70°C and 95°C, was generated to confirm that a single peak was produced for each primer set. Standard curves, produced with serially diluted product, confirmed PCR efficiency of 85% or greater. Size of PCR product was confirmed by electrophoresis on 2% agarose gel. The cycle threshold was measured, with Cq determination mode set to regression. Relative expression was determined using the method described by Pfaffl [26], normalized to two stable reference genes—glyceraldehyde-3-phosphate dehydrogenase (GAPDH) and TATA-binding protein (TBP). For quantification of intracellular DENV copy number, a similar methodology was used. Reverse transcription was performed using M-MuLV reverse transcriptase with random hexamers (New England BioLabs, Ipswich, MA). Quantitative real-time PCR was performed on a Rotor-Gene 6000 (Corbett Life Science, Melbourne, Australia). The DENV RNA copy number was calculated from a standard curve of Mon601 strain DENV-2 that was generated in a parallel PCR and normalized against peptidylprolyl isomerase A (PPIA). Primer sequences for all transcripts are presented in Table 1.

### 2.6. Secreted Interferon-Beta Immunoassay

Culture supernatants from DENV- or mock-infected ARPE-19 cells retinal pigment epithelial cells and retinal endothelial cells were assayed for human IFN-*β* with an AlphaLISA (PerkinElmer, Waltham, MA), with a limit of detection of 1 ng/mL. Human IFN-*β* standard solutions were prepared in medium matched to cell population. The AlphaLISA was performed following the manufacturer's “Quick” protocol exactly, with 2 *μ*L standard or culture supernatant used in each 20 *μ*L assay. Protein concentration was calculated from the AlphaLISA signal read on an EnSight plate reader (PerkinElmer).

### 2.7. Retinal Endothelial VCAM-1 Immunoassay

The cellular adhesion molecule ELISA was adapted from a protocol published by Hartwig et al. [27], as we have previously described [28]. At 48 hours after inoculation with DENV, confluent monolayers of retinal endothelial cells in wells of 96-well plates, infected with DENV-2 or mock infected, were washed twice in PBS with 0.1% Tween-20 (PBS/Tween-20), fixed in 1% paraformaldehyde for 30 minutes, and washed in PBS/Tween-20. Following a 30-minute block with 5% *w*/*v* skim milk in PBS (blocking solution), the monolayers were incubated with mouse monoclonal anti-human vascular cell adhesion molecule 1 (VCAM-1) antibody (BD Pharmingen: catalogue number 555645) or mouse monoclonal IgG (BD Pharmingen) at 1 *μ*g/mL in blocking solution for 45 minutes at room temperature (*n* = 8 monolayers/condition) and washed 5 times with PBS/Tween-20. Subsequently, monolayers were incubated with Alexa Fluor 488-conjugated secondary goat anti-human immunoglobulin antibody at 2.5 *μ*g/mL in blocking solution for 30 minutes at room temperature and washed 5 times with PBS/Tween-20. Finally, monolayers were treated with 300 nM DAPI (Sigma-Aldrich) in PBS for 5 minutes and washed 3 times in PBS. All treatments after the fixation step were performed on a slowly rotating orbital shaker. Monolayer fluorescence was determined on the VICTOR X3 Multilabel Plate Reader (Perkin Elmer) using 485 excitation and 535 emission (Alexa Fluor 488) filters and 355 excitation and 460 emission (DAPI) filters. Mean background fluorescence was determined from mouse IgG-incubated wells for each condition, and this value was subtracted from wells incubated with anti-human VCAM-1 antibody, for the matching condition. Alexa Fluor 488 readings were adjusted for DAPI readings from the same wells to account for differences in cell numbers between wells.

### 2.8. Monolayer Permeability Assay

ARPE-19 retinal pigment epithelial cells or retinal endothelial cells were seeded at 20,000 cells per well in xCELLigence E-Plate 16 (well diameter = 5 mm) (ACEA Biosciences, San Diego, CA) in modified DMEM : F12 or MCDB-131 medium, respectively. After a 2-hour period of recovery, plates were incubated overnight in an xCELLigence RTCA instrument (ACEA Biosciences, San Diego, CA), which maintained the cell monolayers at 37°C and 5% CO_2_ in air, while reading electrical impedance across the monolayers every hour. Plates were temporarily removed from the instrument for addition of DENV in FBS-free medium at MOI of 1, and again temporarily removed from the instrument 90 minutes later, for removal of the DENV suspension and replacement with fresh FBS-supplemented medium. Subsequently, the plates were incubated for approximately 60 hours.

### 2.9. Intracellular Actin Labeling

Confluent monolayers of ARPE-19 retinal pigment epithelial cells or retinal endothelial cells on gelatin-coated glass coverslips were fixed and stored, and immediately prior to labeling, washed and permeabilized, as described above for immunocytochemistry. Cells were labeled with TRITC-tagged phalloidin (Sigma) at 1 *μ*g/mL in PBS with 2% goat serum for 30 minutes at room temperature. Cells were washed in PBS and counterstained with Hoechst 33342 nucleic acid stain for 10 minutes. Monolayers were mounted in ProLong Gold Antifade Mountant and imaged by confocal microscopy.

### 2.10. Statistical Analysis

Data generated under DENV- and mock-infected conditions were compared by unpaired or paired Student's *t*-tests, using CFX Manager version 3.1 (Bio-Rad) or GraphPad Prism v6.04 (GraphPad Software, La Jolla, CA). In all analyses, a significant difference was defined as one yielding a *p* value less than 0.05.

## 3. Results

### 3.1. Human Retinal Pigment Epithelial Cells and Human Retinal Endothelial Cells Are Permissive to DENV Infection

**Figure 1:**
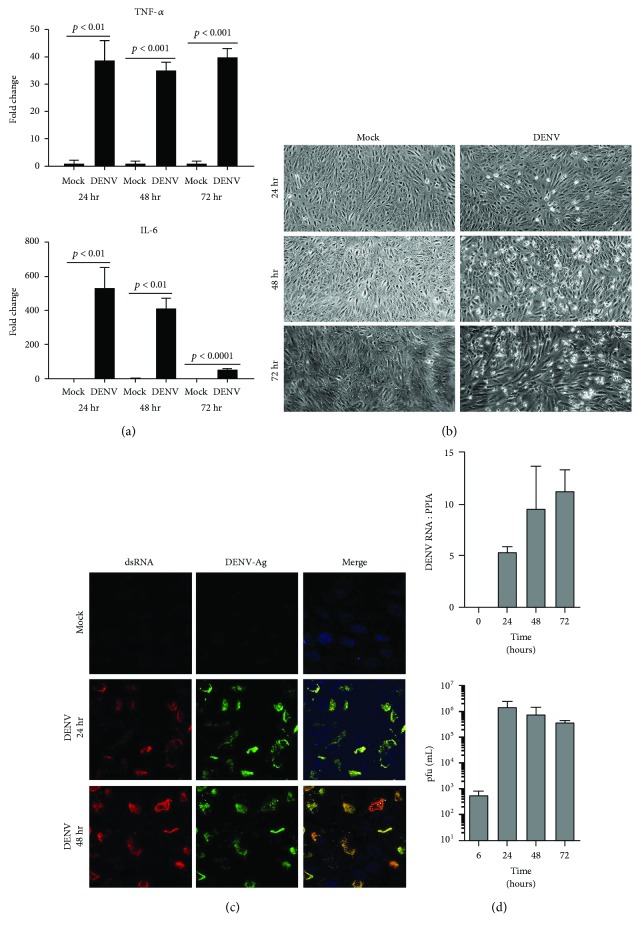
Infection of human retinal pigment epithelial cells with DENV: viral strain = Mon601; multiplicity of infection = 1; evaluated time points postinoculation = 6, 24, 48, and 72 hours (hr). (a) Graphs showing expression of tumor necrosis factor-*α* (TNF-*α*) and interleukin-6 (IL-6) transcripts in DENV-infected retinal pigment epithelial cells versus mock-infected cells. Reference genes were glyceraldehyde-3-phosphate dehydrogenase and TATA-binding protein. Bars represent mean relative expression, with error bars showing standard deviation. *n* = 3 cultures/condition. Data were analyzed by two-tailed Student's *t*-test. (b) DENV-infected and mock-infected retinal pigment epithelial cells viewed by light microscopy. Original magnification = 100x. (c) DENV- and mock-infected retinal pigment epithelial cells immunolabeled to detect double-stranded RNA (dsRNA) and DENV antigen (Ag). Alexa Fluor 555 (red) and Alexa Fluor 488 (green) with Hoechst 33342 nuclear counterstain (blue). Original magnification: 630x. (d) Graphs of DENV RNA copy number for DENV-infected retinal pigment epithelial monolayers and plaque-forming units (pfu) for culture supernatant collected from infected cells. *n* = 3 cultures/condition. Bars represent mean DENV RNA copy number (relative to peptidylprolyl isomerase A (PPIA)) or pfu/mL, with error bars showing standard deviation.

**Figure 2:**
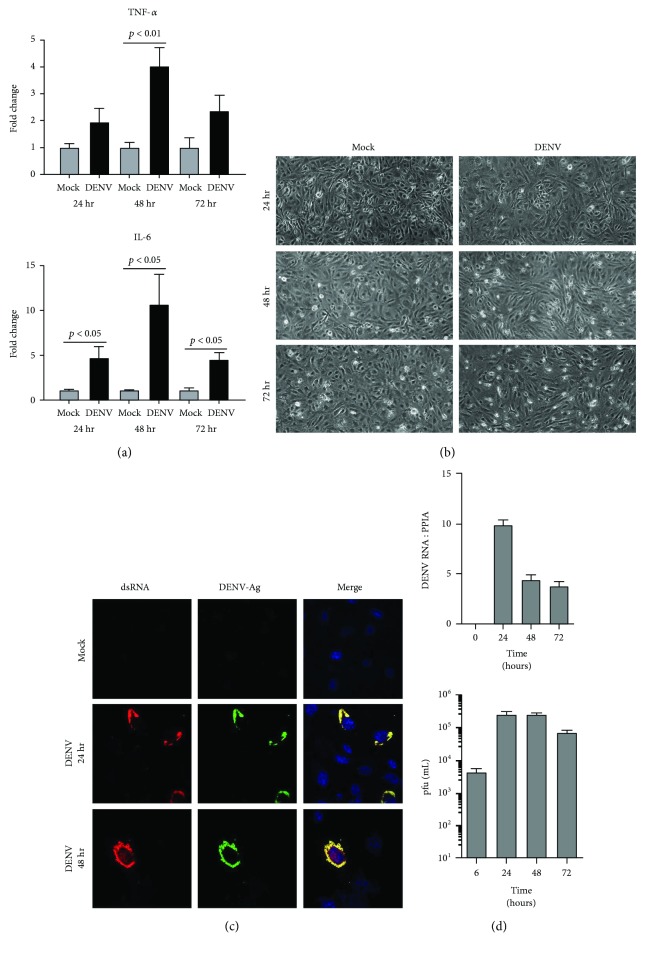
Infection of human retinal endothelial cells with DENV: viral strain = Mon601; multiplicity of infection = 1; evaluated time points postinoculation = 6, 24, 48, and 72 hours (hr). (a) Graphs showing relative expression of tumor necrosis factor-*α* (TNF-*α*) and interleukin-6 (IL-6) transcripts in DENV-infected endothelial cells versus mock-infected cells. Reference genes were glyceraldehyde-3-phosphate dehydrogenase and TATA-binding protein. Bars represent mean relative expression, with error bars showing standard deviation. *n* = 3 cultures/condition. Data were analyzed by two-tailed Student's *t*-test. (b) DENV-infected and mock-infected endothelial cells viewed by light microscopy. Original magnification = 100x. (c) DENV- and mock-infected endothelial cells immunolabeled to detect double-stranded RNA (dsRNA) and DENV antigen (Ag). Alexa Fluor 555 (red) and Alexa Fluor 488 (green) and with Hoechst 33342 nuclear counterstain (blue). Original magnification: 630x. (d) Graphs of copy number of DENV RNA for DENV-infected endothelial monolayers and plaque-forming units (pfu) for culture supernatant collected from infected cells. *n* = 3 cultures/condition. Bars represent DENV RNA copy number (relative to cellular peptidylprolyl isomerase A (PPIA)) or mean pfu/mL, with error bars showing standard deviation.

**Figure 3:**
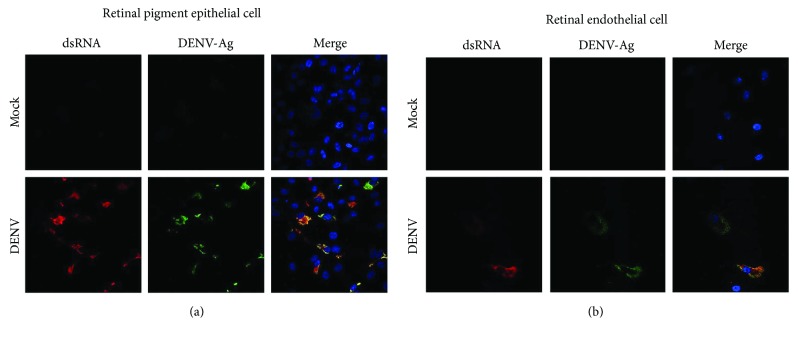
Infection of primary human retinal pigment epithelial cells and primary human retinal endothelial cells with DENV: viral strain = Mon601; multiplicity of infection = 1; evaluated time point postinoculation = 48 hours. (a) DENV- and mock-infected retinal pigment epithelial cells and (b) DENV- and mock-infected retinal endothelial cells immunolabeled to detect double-stranded RNA (dsRNA) and DENV antigen (Ag). Alexa Fluor 555 (red) and Alexa Fluor 488 (green) with Hoechst 33342 nuclear counterstain (blue). Original magnification: 630x.

To initiate the investigation of interactions between DENV, and human retinal pigment epithelial cells and human retinal endothelial cells, we first evaluated the susceptibility of these cells to infection with the virus. We followed the course of DENV-2 infection in ARPE-19 retinal pigment epithelial cells (Figure 1) and in retinal endothelial cells (Figure 2) up to 72 hours by standard cellular and molecular methods. Within 24 hours of inoculation with the virus, both human retinal cell populations demonstrated increased transcription of tumor necrosis factor-*α* (TNF-*α*) and interleukin-6 (IL-6) (Figures 1(a) and 2(a)), which are key cytokines in the development of intraocular inflammation. For pigment epithelial cells, cytopathic effect of the infection was clearly apparent by 48 hours (Figure 1(b)). Immunocytochemistry identified dsRNA and DENV-Ag in a majority of pigment epithelial cells (Figure 1(c)), and RT-qPCR detected a high copy number of DENV-2 RNA in these cells (Figure 1(d)). In contrast, for endothelial cells, there was no obvious cytopathic effect (Figure 2(b)); relatively few cells were positively labeled for dsRNA and DENV-Ag (Figure 2(c)); and RT-qPCR detected a substantially lower copy number of DENV-2 RNA (Figure 2(d)). Analysis of culture supernatant by plaque assay indicated release of infectious virus from both cell types, although viral titer was higher in supernatant harvested from pigment epithelial monolayers than that from endothelial monolayers (Figures 1(d) and 2(d), resp.). We also verified susceptibility of cell populations to infection with DENV-2 by inoculating primary retinal pigment epithelial cells and primary retinal endothelial cells isolated from human cadaver donor eyes. Immunocytochemistry identified dsRNA and DENV-Ag in a majority of primary retinal pigment epithelial cells, but relatively few primary retinal endothelial cells after 48 hours (Figure 3). Taken together, these data suggest both human retinal pigment epithelial cells and human retinal endothelial cells support DENV infection, although the course of the infection may differ between the cell populations.

### 3.2. DENV Infection Induces a Stronger Type I Interferon Response by Human Retinal Pigment Epithelial Cells Than Human Retinal Endothelial Cells

**Figure 4:**
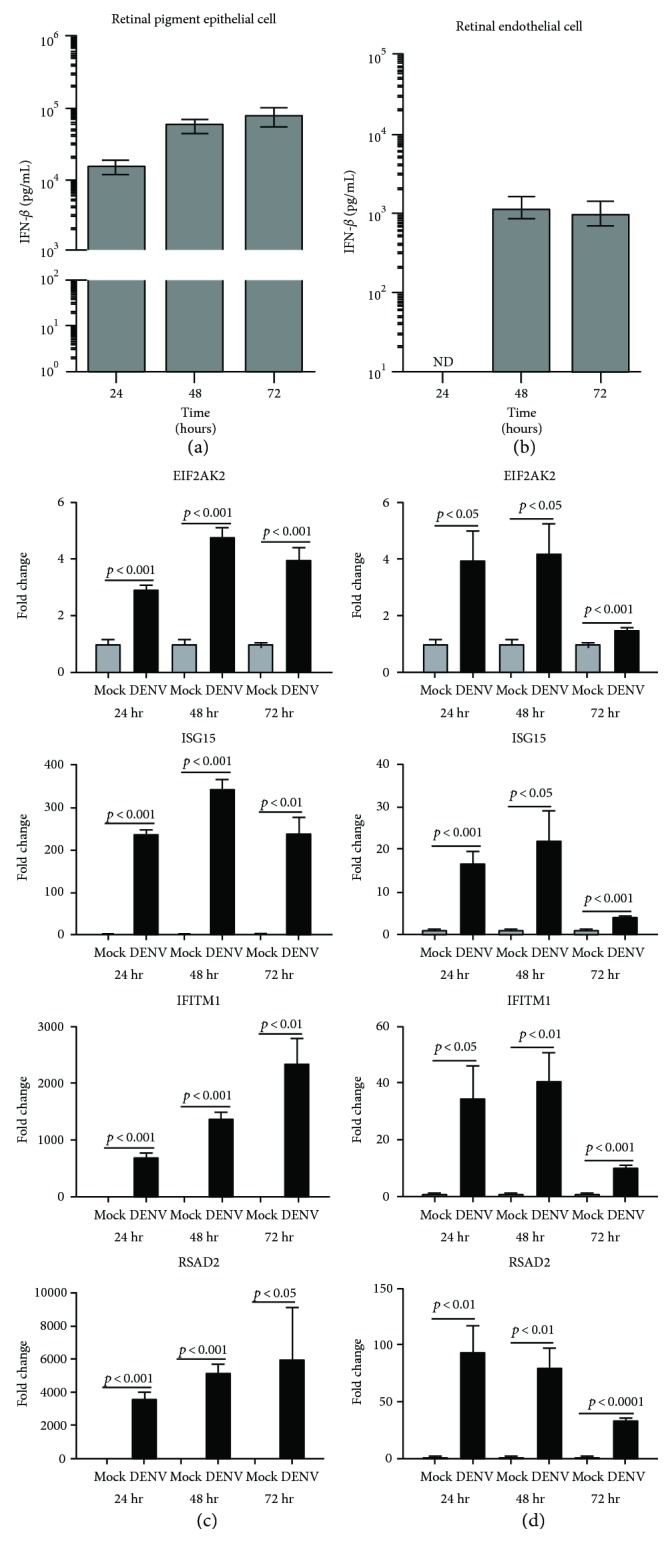
Antiviral type I interferon (IFN) response of human retinal pigment epithelial cells and human retinal endothelial cells infected with DENV: viral strain = Mon601; multiplicity of infection = 1; evaluated time points postinoculation = 24, 48, and 72 hours (hr). (a) and (b) Graphs showing IFN-*β* protein concentration in culture supernatant of (a) DENV-infected retinal pigment epithelial cells and (b) DENV-infected endothelial cells. Bars represent mean protein concentration, with error bars showing standard deviation. *n* = 3 cultures/condition. No IFN-*β* protein was detected in mock-infected cells. ND = not detectable. (c) and (d) Graphs showing relative transcript expression for IFN-stimulated gene products in (c) DENV-infected versus mock-infected retinal pigment epithelial cells and (d) DENV-infected versus mock-infected endothelial cells. Reference genes were glyceraldehyde-3-phosphate dehydrogenase and TATA-binding protein. Bars represent mean relative expression, with error bars showing standard deviation. *n* = 3 cultures/condition. Data were analyzed by two-tailed Student's *t*-test. IFITM1 = IFN-induced transmembrane protein 1; EIF2AK2 = eukaryotic translation initiation factor 2-alpha kinase 2; RSAD2 = radical SAM domain-containing 2 (also known as viperin); ISG15 = IFN-stimulated gene 15.

The type I IFN response is the primary antiviral mechanism effected by human cells [29]. To investigate this response in human retinal pigment epithelial cells and human retinal endothelial cells, we measured IFN-*β* protein in culture supernatant collected from DENV-2-infected and mock-infected cells at 24, 48, and 72 hours postinoculation, using an immunoassay, and we studied induction of transcripts encoding selected IFN-stimulated genes (ISGs) by RT-qPCR at the same time points (Figure 4). Mock-infected cells produced no IFN-*β* protein, to a detection limit of 1 ng/mL. Following infection, however, ARPE-19 retinal pigment epithelial cells secreted IFN-*β* in concentrations exceeding 10 ng/mL at all time points (Figure 4(a)). Infected retinal endothelial cells also secreted IFN-*β*, but the cytokine was not detectable until 48 hours, and concentration was substantially lower, close to the detection limit of the assay (Figure 4(b)). Both cell populations upregulated ISGs in response to viral infection, including factors that inhibit viral entry into the host cell (IFN-induced transmembrane protein 1 (IFITM1)), intracellular replication of virus (eukaryotic translation initiation factor 2-alpha kinase 2 (EIF2AK2) and radical SAM domain-containing 2 (RSAD2), also known as viperin), and viral egress from the cell (IFN-stimulated gene 15 (ISG15)) (Figures 4(c) and 4(d), resp.). These results indicate that human retinal pigment epithelial cells and human retinal endothelial cells both mount an antiviral type I IFN response, but they suggest that this response is more vigorous in the epithelial cells.

### 3.3. DENV Infection Impacts Expression of Cell Adhesion Molecules by Human Retinal Endothelium

**Figure 5:**
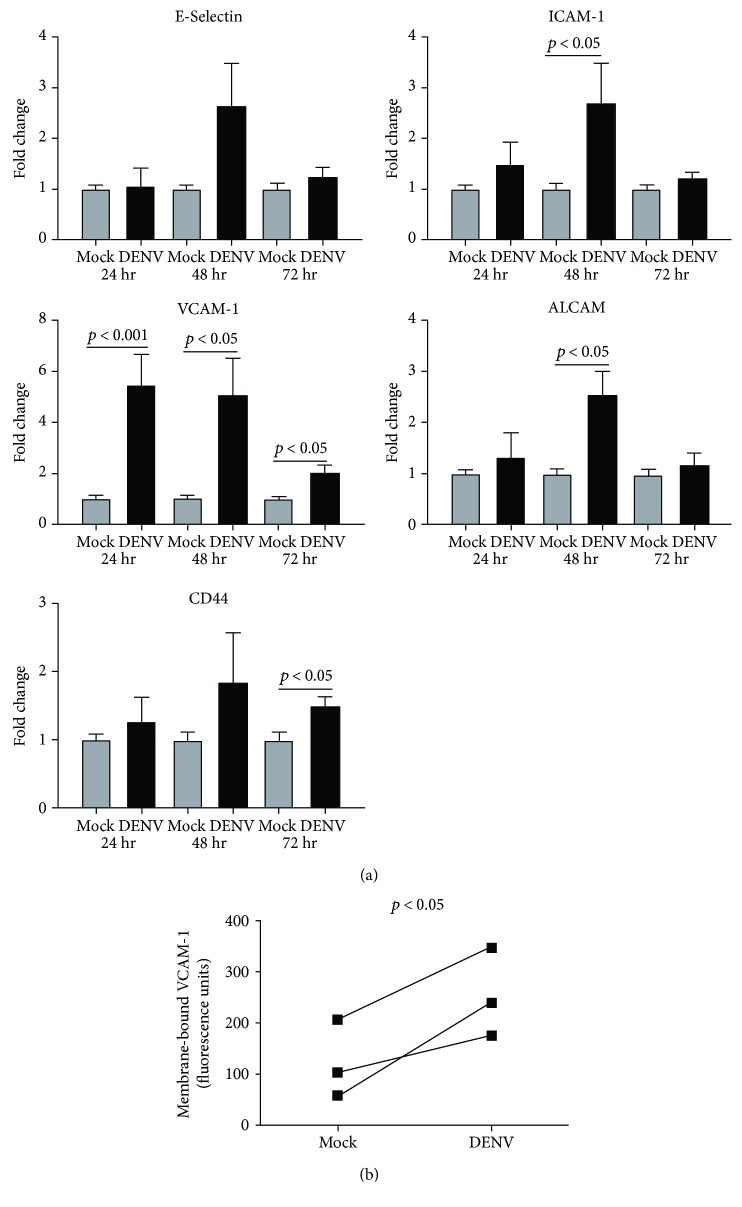
Cell adhesion molecule expression by human retinal endothelial cells infected with DENV: viral strain = Mon601; multiplicity of infection = 1; evaluated time points postinoculation = 24, 48, and 72 hours (hr). (a) Graphs showing relative transcript expression for adhesion molecules in DENV-infected versus mock-infected endothelial cells. Reference genes were glyceraldehyde-3-phosphate dehydrogenase and TATA-binding protein. Bars represent mean relative expression, with error bars showing standard deviation. *n* = 3 cultures/condition. Data were analyzed by two-tailed Student's *t*-test. (b) Graph showing relative expression of VCAM-1 protein on the surface of DENV-infected versus mock-infected endothelial cells. Each pair of circles joined by a line represents the mean expression for DENV-infected versus mock-infected endothelial cells for one of 3 independent experiments. *n* = 8 cultures/condition. Data were analyzed by one-tailed paired Student's *t*-test. ICAM-1 = intercellular adhesion molecule, 1; VCAM-1 = vascular cell adhesion molecule 1; ALCAM = activated leukocyte cell adhesion molecule.

Retinal vasculopathy characterized by retinal vasculitis—inflammation of the retinal blood vessels—is a common manifestation of dengue retinopathy [6]. Human retinal endothelial cells express high levels of adhesion molecules, and expression is further induced under conditions of inflammation, which promotes leukocyte migration into the retina [17]. We examined transcript levels of highly expressed retinal endothelial cell adhesion molecules at 24, 48, and 72 hours postinoculation with DENV-2: E-selectin, intercellular adhesion molecule 1 (ICAM-1), VCAM-1, activated leukocyte cell adhesion molecule (ALCAM), and CD44 (Figure 5(a)). Viral infection led to significant increase in endothelial transcript expression of all adhesion molecules, with the exception of E-selectin, at one or more tested time intervals. Fold increase was highest for VCAM-1 transcript, and this was sustained across 72 hours, suggesting a key role for VCAM-1 in particular, in leukocyte trafficking to the retina in dengue retinopathy. Therefore, we confirmed expression of VCAM-1 with an immunoassay that detected cell membrane-bound adhesion molecule on retinal endothelial monolayers at 48 hours postinoculation: VCAM-1 was significantly increased on DENV-2-infected retinal endothelial cells, in comparison to mock-infected cells (Figure 5(b)).

### 3.4. DENV Infection Promotes Permeability of Human Retinal Pigment Epithelial Monolayers, but Not Human Retinal Endothelial Monolayers, Accompanied by Decreased Expression of Junctional Molecules

**Figure 6:**
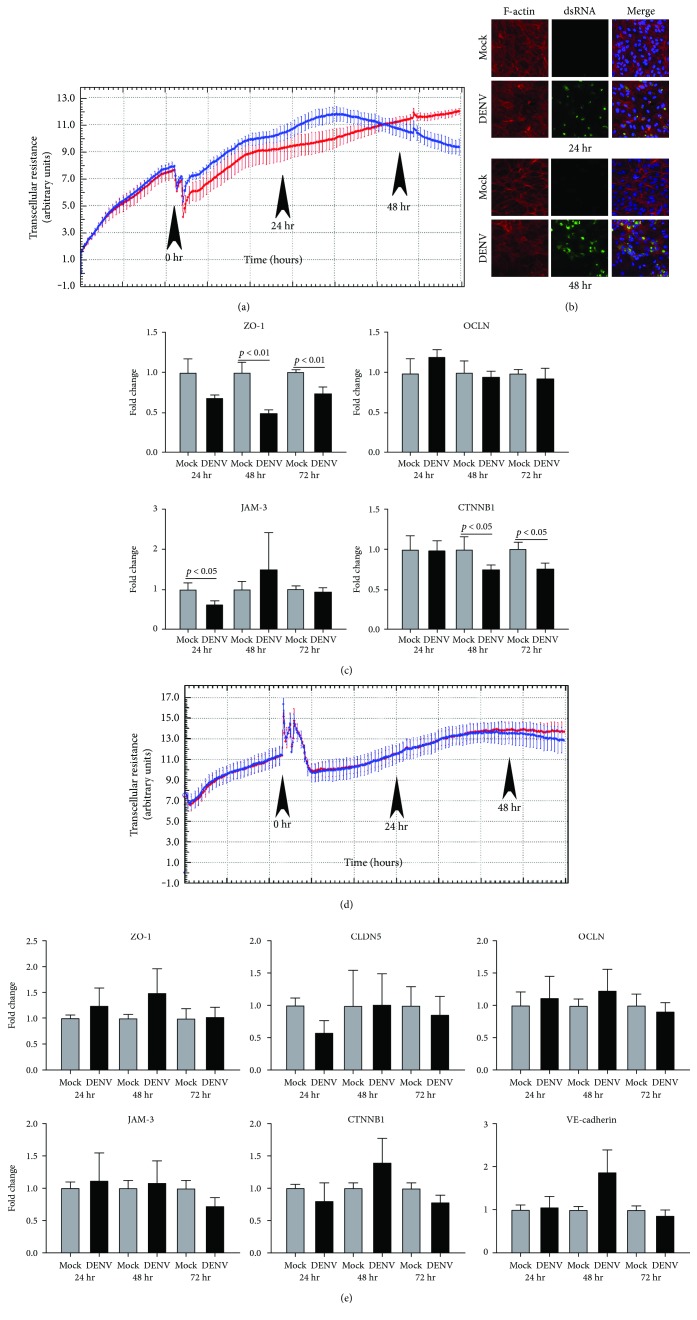
Effect of DENV infection on barrier function of human retinal pigment epithelial cells and human retinal endothelial cells: viral strain = Mon601; multiplicity of infection = 1; evaluated up to 60 hours (hr) postinoculation. (a) and (d) Plots of electrical resistance across (a) DENV-infected retinal pigment epithelial monolayers and (d) DENV-infected endothelial monolayers versus mock-infected monolayers, measured at hourly intervals and expressed as transcellular resistance by xCELLigence. Lines indicate mean electrical resistance: blue lines represent DENV-infected monolayers and red lines represent mock-infected monolayers. Error bars indicate standard error of mean. *n* = 4 monolayers/condition. Arrowheads mark time of inoculation and 24 and 48 hours postinoculation. Drop in resistance immediately after inoculation with virus is due to removal of plate from xCELLigence for inoculation procedure. (b) DENV- and mock-infected retinal pigment epithelial cells immunolabeled to detect double-stranded RNA (dsRNA) and filamentous- (F-) actin. Alexa Fluor 555 (red) and Alexa Fluor 488 (green) with Hoechst 33342 nuclear counterstain (blue). Images are merged z-stacks. Original magnification: 630x. (c) and (e) Graphs showing relative transcript expression for junctional molecules in (c) DENV-infected retinal pigment epithelial cells and (e) DENV-infected endothelial cells versus mock-infected cells. Reference genes were glyceraldehyde-3-phosphate dehydrogenase and TATA-binding protein. Bars represent mean relative expression, with error bars showing standard deviation. *n* = 3 cultures/condition. Data were analyzed by two-tailed Student's *t*-test. ZO-1 = zonula occludens 1; CLDN5 = claudin 5; OCLN = occludin; JAM-3 = junctional adhesion molecule 3; CTNNB1 = catenin-*β*1. CLDN5 and VE-cadherin were not detected in epithelial cells.

Since excessive accumulation of extracellular fluid in the macular region disturbs neuroretinal anatomy, macular edema carries the greatest risk to the vision in dengue retinopathy [6]. The blood-retinal barrier is formed by the retinal pigment epithelium, which abuts the highly fenestrated choroidal vasculature and the retinal vascular endothelium [30]. We evaluated the impact of DENV-2 infection on barrier function and phenotype of these cells (Figure 6). Effect on barrier function was assessed in real time by measuring transcellular electrical resistance every hour over a period of 3 days. Although electrical resistance across ARPE-19 retinal pigment epithelial monolayers infected with DENV-2 was higher than across mock-infected cells, at approximately 36 hours postinoculation, electrical resistance began to decline across the infected monolayers, while it continued to climb across mock-infected cells (Figure 6(a)). To independently confirm this alteration, we examined the filamentous- (F-) actin arrangement by phalloidin staining cells at 24 and 48 hours postinoculation. In mock-infected cells, a well-organized intracellular network of filaments was maintained, whereas in DENV-2-infected cells, disorganization of the network, with aggregations and loss of filaments colocalizing with areas of viral replication, was observed by 24 hours (Figure 6(b)). To determine the molecular basis for loss of barrier function, we examined the expression of transcripts encoding molecules involved in epithelial adherens and tight junctions at 24, 48, and 72 hours postinoculation by RT-qPCR: expression of zonula occludens 1 (ZO-1), junctional adhesion molecule 3 (JAM-3), and/or catenin-*β*1 (CTNNB1) was decreased across these intervals (Figure 6(c)). In contrast to changes in electrical resistance observed in DENV-2-infected retinal pigment epithelial monolayers, electrical resistance measured across retinal endothelial monolayers was comparable between DENV-2-infected and mock-infected cells, rising over approximately 48 hours before stabilizing (Figure 6(d)). Consistently, the expression of transcripts encoding molecules involved in endothelial adherens and tight junctions, including endothelial-specific VE-cadherin, were not impacted by infection (Figure 6(e)). Taken together, these data suggest breakdown of the blood-retinal barrier during dengue retinopathy occurs at the level of the retinal pigment epithelium.

### 3.5. Response of Human Retinal Cells to DENV Infection Is Replicated with Natural Isolate Strain

Our studies were performed with the laboratory-cloned Mon601 DENV-2 strain. To confirm the relevance of our findings to the human infectious disease, we repeated selected infectivity and host cell response experiments using PUO-312, which is a DENV-2 strain isolated from an infected human [21, 22] (Figure S1 available online at https://doi.org/10.1155/2017/3164375). Experiments were analyzed at 48 hours postinoculation with PUO-312, which was the time at which we observed most marked pathological changes in experiments with Mon601. Immunocytochemistry identified dsRNA and DENV-Ag in PUO-312-infected ARPE-19 retinal pigment epithelial cells and retinal endothelial cells, but not mock-infected cells (Figure S1 A and B, resp.). Infection with PUO-312 resulted in similar changes in transcript expression to those observed with the laboratory clone: increased TNF-*α* in retinal pigment epithelial cells and retinal endothelial cells (not statistically significant for endothelial cells); increased IFN-*β* in retinal pigment epithelial cells and retinal endothelial cells; increased VCAM-1 in retinal endothelial cells; and decreased CTNNB1 in retinal pigment epithelial cells, but not retinal endothelial cells (Figure S1 C and D, resp.). These results provide evidence that observations made in experiments using the Mon601 are representative of cellular and molecular responses to DENV strains responsible for disease in humans.

## 4. Discussion

Dengue retinopathy is the most serious ocular involvement that follows systemic infection with DENV [6]. Data collected during the 2005 Singapore epidemic [12] indicate that this condition may affect as many as 10% of patients who require hospitalization for DENV infection. There is no antiviral drug that specifically targets DENV, and in the absence of studies of disease pathogenesis, treatment of dengue retinopathy is limited to reducing the host inflammatory response nonspecifically by treatment with locally or systemically delivered corticosteroid and/or intravenous immunoglobulin [10, 11]. We have conducted a series of experiments designed to investigate basic mechanisms operating during DENV retinopathy, based on multiple clinical observations that implicate cells of the blood-retinal barrier, retinal pigment epithelial cells and retinal endothelial cells, in the ocular pathology [7–13].

Infectivity studies, including immunolabeling of viral dsRNA and DENV antigen in cultured cells, RT-qPCR of DENV transcript in cells, and plaque assay of cell culture supernatant, all indicated that retinal pigment epithelial cells and retinal endothelial cells were permissive to viral infection and supported release of infectious virus. Infection of human retinal cells with DENV has not been studied previously, but these retinal cell populations are reported to be susceptible to infection with other RNA and DNA viruses [31–35]. Work performed by ourselves and independent researchers [23, 36, 37] has highlighted that nonocular endothelial cell populations, including endothelial cells from the human umbilical vein and the human dermal microvasculature, are susceptible to infection with DENV, but with a relatively low proportion of cells infected; this has been attributed to both extracellular conditions and cellular phenotype, including the expression of cell surface receptors for the virus. Consistently, we observed that human retinal pigment epithelial cells showed evidence of the viral cytopathic effect and were infected in larger numbers, contained higher viral copy number, and released virus at higher titer than human retinal endothelial cells. The pigment epithelial cells also reacted to infection with higher production of IFN-*β*, although both pigment epithelial cells and endothelial cells types effected a type-1 IFN response.

The human retina may manifest pathology after systemic infection with a diverse spectrum of RNA and DNA viruses [38]. Clinical features of viral retinopathy vary with the pathogen, which may reflect factors such as route of viral entry into the eye, specific retinal cell tropism of the virus, retinal host cell response to the virus, and the host immune response to the infection. In addition to DENV, two other flaviviruses—West Nile virus and Zika virus—may cause retinopathy, with characteristic forms that differ from that of dengue retinopathy. West Nile virus infection typically causes multifocal chorioretinal lesions that occur in linear streaks following the course of retinal nerve fibers [39, 40]. Zika virus retinopathy is described mostly in infants born with microcephaly, who were infected in utero; affected infants suffer pigment mottling of the retina and large areas of chorioretinal atrophy that often are located at the macula and shaped like a torpedo [41, 42]. Recently, other groups have described responses of several retinal cell populations to infection with West Nile virus or Zika virus [31, 33, 43]. Our work is directed at understanding dengue retinopathy in particular—retinal vasculopathy and maculopathy—at the cellular and molecular level.

Retinal vasculitis is a prominent clinical feature of dengue retinopathy and may be associated with retinal hemorrhages and/or vascular occlusions [6]. At the molecular level, cell adhesion molecules on activated retinal vascular endothelium mediate interactions with myeloid and lymphoid leukocyte subsets that permit leukocyte transendothelial migration [17]. Studies of noninfectious retinal inflammation, utilizing mouse experimental autoimmune uveoretinitis or human simulations, have implicated integrins, superfamily immunoglobulin members, and/or CD44 in leukocyte trafficking into the retina [44–47]. We measured increased expression of ICAM-1, VCAM-1, ALCAM, and CD44 transcript by infected human retinal endothelial cells at different intervals after DENV infection. Increased expression of VCAM-1 was sustained across all intervals that we studied, and this was confirmed by detection of increased VCAM-1 protein on the surface of DENV-infected cells. A key role for VCAM-1 in the pathogenesis of dengue retinopathy is corroborated by several recent studies of systemic biomarkers in persons infected with DENV. Yacoub et al. [48] linked plasma levels of cleaved VCAM-1, but not E-selectin or ICAM-1, with parameters of microcirculatory dysfunction in patients with serologically confirmed dengue of any DENV serotype. Liao et al. [49] reported soluble VCAM-1 to be an early biomarker of severe dengue versus dengue fever in adults infected with DENV-1, and Mangione et al. [50] similarly associated soluble VCAM-1 with pediatric dengue shock syndrome, in comparison to dengue fever and dengue hemorrhagic fever.

Maculopathy is the other common manifestation of dengue retinopathy, usually presenting as macular edema and implying breakdown of the blood-retinal barrier. Velandia-Romero et al. [51] demonstrated the ability of DENV to bridge the vascular-tissue barrier in a mouse *in vitro* model, in which transwells were populated with mouse brain endothelial cells, and transendothelial potential was measured four times daily for 2 days: movement of virus was accompanied by an increased permeability of the monolayer that was partially abrogated by co-culture of the endothelial cells with mouse astrocytes. The blood-retinal barrier is constituted at two anatomically separate locations, by the nonfenestrated retinal vascular endothelium and the retinal pigment epithelium [30]. We observed increasing permeability of infected retinal pigment epithelial monolayers, in comparison to retinal endothelial monolayers, using a closed system with hourly electrical impedance readings over 3 days. This response of the retinal pigment epithelium was accompanied by loss of cellular F-actin structure and a reduced expression of junctional molecules that was not recorded for human retinal endothelial cells. This suggests macular edema may reflect primarily the infection of retinal pigment epithelium. Involvement of the retinal pigment epithelium is also consistent with the less common, but highly characteristic, clinical finding of foveolitis, which occurs at the junction of photoreceptors and epithelium. Interestingly, in a model of retinal infection with a parasite, *Toxoplasma gondii*, which used the ARPE-19 retinal pigment epithelial cells we employed, disruption of the retinal pigment epithelium was similarly documented [52].

This investigation focuses on interactions between DENV and the cells of the blood-retinal barrier. Another aspect of the pathogenesis of dengue retinopathy is the interaction between DENV-infected retinal pigment epithelial cells or retinal endothelial cells, as well as other retinal cell populations, and the infiltrating leukocytes, which also may be infected. Special immunological features of pigment epithelial cells and endothelial cells influence the course of a retinal inflammation. Retinal pigment epithelial cells produce a range of immunomodulatory molecules that act to downregulate inflammatory activities of leukocyte populations [53]. On the other hand, retinal endothelial cells constitutively express relatively high levels of cell adhesion molecules and chemokines, and they have the capacity to produce a variety of other molecules that may act to promote inflammation [54]. Studies of leukocyte-retinal cell interactions should further illuminate the mechanisms of dengue retinopathy.

An important question is the impact of an individual viral phenotype on interactions with the host cell. Our primary goal in this work was to initiate an investigation into the basic mechanisms of dengue retinopathy. Therefore, for these experiments, we selected the Mon601 strain, which is widely used in studies of DENV infection, being pathogenic and one of the first established as a reliable molecular clone [20]. The comparison of two dengue epidemics in Singapore, in which different DENV serotypes predominated, yielded different prevalences for dengue maculopathy, suggesting viral phenotype may impact clinical manifestations [55]. Serotype and disease associations are not straightforward to identify, however, since although one serotype may predominate during an epidemic, the infecting serotypes are often not defined, and in addition to serotype associations, there also may be strain associations. A comprehensive investigation of the effect of viral phenotype in dengue retinopathy will need to consider not only serotypes 1 through 4, but also multiple strains of each of the 4 serotypes.

In summary, we have demonstrated that human retinal pigment epithelial cells and human retinal endothelial cells are permissive to infection with DENV, and we have provided molecular correlations for common manifestations of dengue retinopathy. Our work was conducted *in vitro*, using an infectious, recombinant DENV-2 clone and characterized human retinal cell lines, and key findings of our study were replicated in primary human retinal cells, and with the PUO-312 strain DENV-2, which was isolated from an infected human. *In vitro* cell responses may not predict the clinical disease *in vivo* in the human host. *In vitro* studies with human retinal cells are helpful for understanding dengue maculopathy in particular, since primates are the only mammals with a macula, and nonhuman primates develop little or no clinical disease following infection with dengue [56]. However, *in vivo* studies in mice are likely to provide mechanistic insights relating to other retinal manifestations; we have detected DENV RNA in eyes of AG129 IFN receptor-deficient mice and C57BL/6 mice following systemic and intracranial DENV-2 infection, respectively, suggesting that both may provide experimental models of dengue eye disease (unpublished data). Cell populations inside the blood-retinal barrier, including retinal neurons, Müller glial cells, astrocytes, microglial cells, and pericytes, also may participate in the pathogenesis of dengue retinopathy; microglia may be important for an anti-DENV immune response, as has been demonstrated in experimental encephalitis [57]. Potential roles of these cells could be explored in both *in vitro* and *in vivo* studies. Recognizing the need for additional research in this new field, the present work provides a solid basis for future studies on the basic mechanisms of dengue retinopathy, which ultimately should allow the development of more specific treatments for this vision-threatening condition.

## 5. Conclusions

Retinopathy, or disease involving the retina, is the most serious eye complication for individuals who become infected with DENV. Our research seeks to explain the features of dengue retinopathy, as seen in patients, at the molecular level. This work focuses on the interaction between DENV and two human retinal cell populations that are implicated in this disease through clinical observations: pathological changes along the retinal blood vessels and abnormal accumulation of extracellular fluid in the central macular region of retina implicate retinal endothelial cells and retinal pigment epithelial cells. We find that both retinal cell populations support the replication and release of DENV, and mount antiviral molecular responses, with some differences in the level of these activities between the cell types. We show that molecules that bind leukocytes are increased in infected retinal endothelial cells, providing a basis for retinal vascular pathology. We also identify alterations in intercellular junctions following infection of pigment epithelial cells, but not endothelial cells, that would promote leakage of extracellular fluid into the retina. Our findings provide clinicopathological correlations in dengue retinopathy and begin to address the possibility of developing biologically relevant treatments for this condition.

## Supplementary Material

Supplementary Figure 1. Infection of human retinal pigment epithelial cells and human retinal endothelial cells with patient DENV-2 isolate: viral strain = PUO-312; multiplicity of infection = 1; evaluated time point post-inoculation = 48 hours. (A) and (B) DENV- and mock-infected (A) retinal pigment epithelial cells and (B) retinal endothelial cells immunolabeled to detect double stranded (ds)RNA and DENV antigen (Ag). Alexa Fluor 555 (red) and Alexa Fluor 488 (green) with Hoechst 33342 nuclear counterstain (blue). Original magnification: 630x. (C) and (D) Graphs showing expression of selected transcripts in DENV-infected (C) retinal pigment epithelial cells and (D) retinal endothelial cells, versus mock-infected cells. Reference genes were glyceraldehyde-3-phosphate dehydrogenase and TATA-binding protein. Bars represent mean relative expression, with error bars showing standard deviation. n = 3 cultures/condition. Data were analyzed by two-tailed Student's *t*-test. TNF-*α* = tumor necrosis factor-*α*, IFN-*β* = interferon-*β*, VCAM-1 = vascular cell adhesion molecule 1, CTNNB1 = catenin-*β*1.

## Figures and Tables

**Table 1:** Primer pairs and product sizes for gene transcripts.

Gene transcript^∗^	Primer pair	Product size (bp)
CLDN5 [58]	Forward: 5′-GCAGCCCCTGTGAAGATTGA-3′ Reverse: 5′-GTCTCTGGCAAAAAGCGGTG-3′	106
CTNNB1 [59]	Forward: 5′-AAAGCGGCTGTTAGTCACTGG-3′ Reverse: 5′-GACTTGGGAGGTATCCACATCC-3′	132
DENV	Forward: 5′-GCAGATCTCTGATGAATAACCAAC-3′ Reverse: 5′-TTGTCAGCTGTTGTACAGTCG-3′	102
EIF2AK2 [60]	Forward: 5′-GGAAAGCGAACAAGGAGTAAGG-3′ Reverse: 5′-CCAAAGCGTAGAGGTCCACT-3′	97
GAPDH	Forward: 5′-AGCTGAACGGGAAGCTCACTGG-3′ Reverse: 5′-GGAGTGGGTGTCGCTGTTGAAGTC-3′	209
IFITM1 [61]	Forward: 5′-ACTCCGTGAAGTCTAGGGACA-3′ Reverse: 5′-TGTCACAGAGCCGAATACCAG-3′	155
IFN-*β* [62]	Forward: 5′-AAACTCATGAGCAGTCTGCA-3′ Reverse: 5′-AGGAGATCTTCAGTTTCGGAGG-3′	168
IL-6 [63]	Forward: 5′-ATGAACTCCTTCTCCACAAGCGC-3′ Reverse: 5′-GAAGAGCCCTCAGGCTGGACTG-3′	628
ISG15 [64]	Forward: 5′-GAGAGGCAGCGAACTCATCT-3′ Reverse: 5′-AGCATCTTCACCGTCAGGTC-3′	99
JAM-3 [65]	Forward: 5′-CCCTGTCTGTAGAGTGCCGAAG-3′ Reverse: 5′-GAGCCTGCGTCATTGGAAGC-3′	258
OCLN [66]	Forward: 5′-TGCATGTTCGACCAATGC-3′ Reverse: 5′-AAGCCACTTCCTCCATAAGG-3′	235
PPIA	Forward: 5′-GGCAAATGCTGGACCCAACACAAA-3′ Reverse: 5′-CTAGGCATGGGAGGGAACAAGGAA-3′	355
RSAD2 [67]	Forward: 5′-TGACGGAACAGATCAAAGCA-3′ Reverse: 5′-GCACCAAGCAGGACACTTCT-3′	174
TBP [68]	Forward: 5′-GCCTCCCCCACCCCCTTCTTT-3′ Reverse: 5′-GCCACACCCTGCAACTCAACATCC-3′	106
TNF-*α* [63]	Forward: 5′-TCTCGAACCCCGAGTGACAA-3′ Reverse: 5′-TGAAGAGGACCTGGGAGTAG-3′	181
VE-cadherin [69]	Forward: 5′-GGGTTTTTGCATAATAAGCAGG-3′ Reverse: 5′-GCACCAGTTTGGCCAATATA-3′	149
ZO-1 [70]	Forward: 5′-GAACGAGGCATCATCCCTAA-3′ Reverse: 5′-CCAGCTTCTCGAAGAACCAC-3′	218

^∗^References for primers sequences sourced from the literature are provided. For primer pairs designed in-house, products were confirmed by sequencing. B2M: *β*-2-microglobulin; CLDN5: claudin 5; CTNNB1: catenin-*β*1; DENV: dengue viral RNA; EIF2AK2: eukaryotic translation initiation factor 2-alpha kinase 2; GAPDH: glyceraldehyde-3-phosphate dehydrogenase; IFITM1: interferon-induced transmembrane protein 1; IFN-*β*: interferon-*β*; IL-6: interleukin-6; ISG15: interferon-stimulated gene 15; JAM-3: junctional adhesion molecule 3; OCLN: occludin; PPIA: peptidylprolyl isomerase A; RSAD2: radical SAM domain-containing 2 (viperin); TBP: TATA-binding protein; TNF-*α*: tumor necrosis factor-*α*; ZO-1: zonula occludens 1.
