# Erratum to “Molecular Responses of Human Retinal Cells to Infection with Dengue Virus”

**DOI:** 10.1155/2018/9891049

**Published:** 2018-12-23

**Authors:** Jillian M. Carr, Liam M. Ashander, Julie K. Calvert, Yuefang Ma, Amanda Aloia, Gustavo G. Bracho, Soon-Phaik Chee, Binoy Appukuttan, Justine R. Smith

**Affiliations:** ^1^Microbiology & Infectious Diseases, Flinders University School of Medicine, Rm 5D-316, 1 Flinders Drive, Bedford Park, Adelaide, SA 5042, Australia; ^2^Eye & Vision Health, Flinders University School of Medicine, Rm 4E-431, 1 Flinders Drive, Bedford Park, Adelaide, SA 5042, Australia; ^3^Flinders Centre for Innovation in Cancer, Flinders University School of Medicine, 1 Flinders Drive, Bedford Park, Adelaide, SA 5042, Australia; ^4^Ocular Inflammation and Immunology Service, 11 Third Hospital Avenue, Singapore National Eye Centre, Singapore 168751

In the article titled “Molecular Responses of Human Retinal Cells to Infection with Dengue Virus,” [1] there was an error in the first graph in Figure 2(d). The numbers on the *y*-axis were incorrect. This error occurred during the production process. The correct figure is shown below.

## Figures and Tables

**Figure 1:**
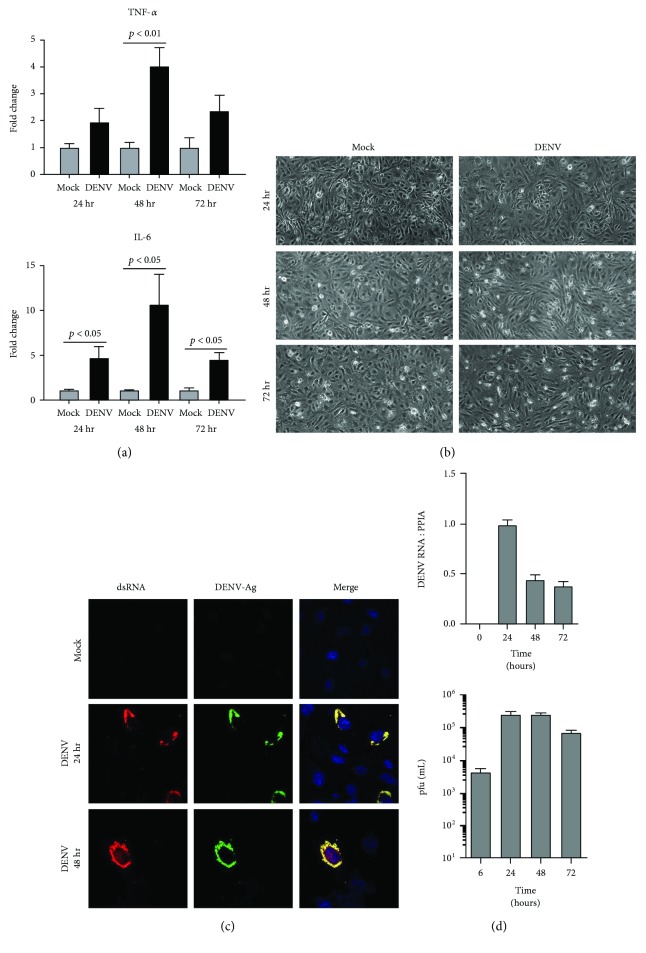
Infection of human retinal endothelial cells with DENV: viral strain = Mon601; multiplicity of infection = 1; evaluated time points postinoculation = 6, 24, 48, and 72 hours (hr). (a) Graphs showing relative expression of tumor necrosis factor-*α* (TNF-*α*) and interleukin-6 (IL-6) transcripts in DENV-infected endothelial cells versus mock-infected cells. Reference genes were glyceraldehyde-3-phosphate dehydrogenase and TATA-binding protein. Bars represent mean relative expression, with error bars showing standard deviation. *n* = 3 cultures/condition. Data were analyzed by two-tailed Student's *t*-test. (b) DENV-infected and mock-infected endothelial cells viewed by light microscopy. Original magnification = 100x. (c) DENV- and mock-infected endothelial cells immunolabeled to detect double-stranded RNA (dsRNA) and DENV antigen (Ag). Alexa Fluor 555 (red) and Alexa Fluor 488 (green) and with Hoechst 33342 nuclear counterstain (blue). Original magnification: 630x. (d) Graphs of copy number of DENV RNA for DENV-infected endothelial monolayers and plaque-forming units (pfu) for culture supernatant collected from infected cells. *n* = 3 cultures/condition. Bars represent DENV RNA copy number (relative to cellular peptidylprolyl isomerase A (PPIA)) or mean pfu/mL, with error bars showing standard deviation.
