# Supplementary figures and images for: Molecular Responses of Human Retinal Cells to Infection with Dengue Virus
Source: Mediators Inflamm. 2017 Nov 12;2017:3164375. doi: 10.1155/2017/3164375 (PMC5829438; doi:10.1155/2017/3164375)

Figure S1

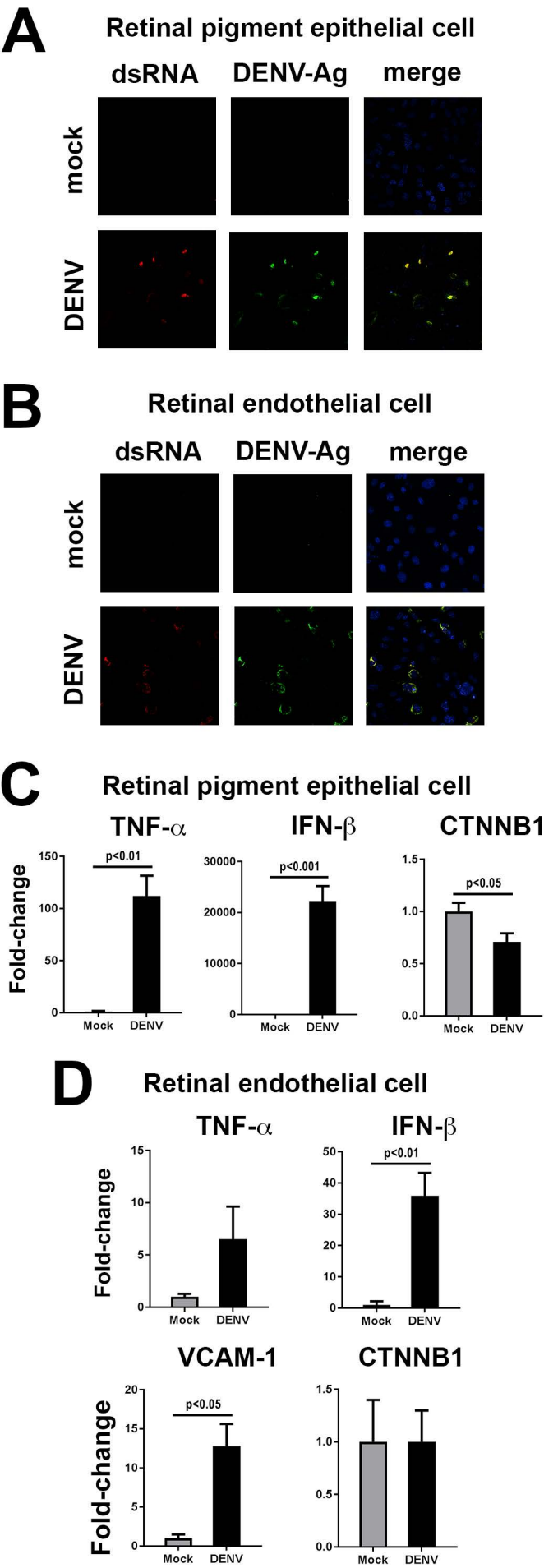

Supplement: Supplementary file 2 [file 3164375.f2.pdf]
